# Automated identification of the mouse brain’s spatial compartments from in situ sequencing data

**DOI:** 10.1186/s12915-020-00874-5

**Published:** 2020-10-19

**Authors:** Gabriele Partel, Markus M. Hilscher, Giorgia Milli, Leslie Solorzano, Anna H. Klemm, Mats Nilsson, Carolina Wählby

**Affiliations:** 1 Centre for Image Analysis, Department of Information Technology and Science for Life Laboratory, Uppsala University, Uppsala, Sweden; 2 Science for Life Laboratory, Department of Biochemistry and Biophysics, Stockholm University, Solna, Sweden; 3 BioImage Informatics Facility of SciLifeLab, Uppsala, Sweden

**Keywords:** In situ sequencing, Spatial analysis, Brain compartments

## Abstract

**Background:**

Neuroanatomical compartments of the mouse brain are identified and outlined mainly based on manual annotations of samples using features related to tissue and cellular morphology, taking advantage of publicly available reference atlases. However, this task is challenging since sliced tissue sections are rarely perfectly parallel or angled with respect to sections in the reference atlas and organs from different individuals may vary in size and shape and requires manual annotation. With the advent of in situ sequencing technologies and automated approaches, it is now possible to profile the gene expression of targeted genes inside preserved tissue samples and thus spatially map biological processes across anatomical compartments.

**Results:**

Here, we show how in situ sequencing data combined with dimensionality reduction and clustering can be used to identify spatial compartments that correspond to known anatomical compartments of the brain. We also visualize gradients in gene expression and sharp as well as smooth transitions between different compartments. We apply our method on mouse brain sections and show that a fully unsupervised approach can computationally define anatomical compartments, which are highly reproducible across individuals, using as few as 18 gene markers. We also show that morphological variation does not always follow gene expression, and different spatial compartments can be defined by various cell types with common morphological features but distinct gene expression profiles.

**Conclusion:**

We show that spatial gene expression data can be used for unsupervised and unbiased annotations of mouse brain spatial compartments based only on molecular markers, without the need of subjective manual annotations based on tissue and cell morphology or matching reference atlases.

## Background

Highly multiplexed spatial expression analysis of genes is essential to uncover the organization of biological processes in relation to sub-regions of tissues and organs. Spatial atlases for well-studied organs like the brain exist [1], but matching individual samples to a reference atlas is challenging as 2D sections may be angled and not well-aligned, and shape and size of organs differ between individuals. Furthermore, specimens from human organs are often sampled from a part of an organ, and it is generally not trivial to identify the exact original location and orientation of the analyzed sample. Some regions, such as the pyramidal cell layer of the hippocampus in the brain, can be well defined based on morphology, whereas other regions are more difficult to identify as the morphological variations are small when cellular labeling is limited to nuclear staining or a finite set of fluorescent markers.

We hypothesize that tissue and cell type can be defined by gene expression and that gene expression thus should define spatial compartments. Several methods for measuring gene expression while preserving spatial information have been developed over the past years. Generally described, there are two approaches to preserve spatial information: One approach is to either imprint a spatial reference on a grid or to carefully record spatial location prior to collection and sequencing of single-cell RNA [2–6]. The other approach is the parallel profiling of large numbers of mRNAs using barcodes decoded directly in the tissue sample [7–10]. All approaches come with benefits and drawbacks, such as spatial resolution, depth of sequencing, accuracy, and throughput [11].

Targeted in situ sequencing (ISS) using padlock probes and localized rolling circle amplification [7] provides submicron localization of RNA species in a highly multiplexed fashion in cells and entire tissue sections, and recent advancements in automation [12] have led to higher detection efficiency and shorter protocol times. Investigated genes are targeted with carefully designed barcoded padlock probes, locally amplified and sequenced by repeated fluorescent staining and imaging cycles. The resulting image data consists of six fluorescent channels for each sequencing cycle: One channel showing cell nuclei, a reference channel for probe location, and four channels (i.e., color channels) with fluorescent signals representing the four bases of the genetic code (A, C, G, T). The resulting fluorescent signals appear as bright spots in a noisy background caused by light scattering and autofluorescence in the tissue.

Previous approaches to define spatial compartments based on exploring spatial patterns are non-negative matrix factorization [13] and SpatialDE [14]. Non-negative matrix factorization is a linear dimensionality reduction technique that can provide a series of locally correlated gene expression maps representing distinct biological processes; local gene correlation does however not necessarily define distinct tissue types. SpatialDE identifies spatially variable genes and clusters their spatial profiles based on a Gaussian-process-based prior. Genes with similar spatial variation are thus grouped together into spatially significant patterns; again, similarity in variation across genes may not correlate with similarity in variation across tissue types.

In this paper, we first decode ISS data using an image analysis pipeline based on a graph-based decoding approach. We detect fluorescent signals applying a generous threshold and then revolve with high precision all targeted barcodes using a graphical model (Methods), thus pushing spatial gene expression profiling of tissue samples to large sample coverage, high multiplexity, and high decoding resolution. We analyze four different coronal brain sections from two different mice, and we further verify that decoded per-gene spatial expression patterns match with in situ hybridization patterns from the Allen Mouse Brain Atlas [1].

Next, we apply dimensionality reduction of local gene expression patterns by UMAP manifold learning [15]. We show how this manifold can be reduced to three dimensions and visualized in RGB color space, providing a general visualization of variations in gene expression. We continue with community-based clustering of the manifold space (Methods), resulting in a novel way of detecting spatial compartments representing unique combinations of expressed genes and cell types. We show that the spatial compartments emerging from these clusters are highly reproducible across brain tissue sections from two different individuals. We also explore the possibility of reducing the number of different genes required for defining unique and reproducible spatial compartments, enabling unbiased differential expression analysis between identified compartments.

We further compare the spatial compartments defined based on gene expression patterns with local cell morphology (using CellProfiler [16]) and clusters defined based on correlation patterns from non-negative matrix factorization and SpatialDE. All these methods detect spatial variations. However, spatial compartments defined from gene expression have higher discriminative power and reproducibility and thus can better guide automated identification of anatomical regions for further investigations in relation to development or disease.

## Results

### In situ sequence decoding reveals symmetric and repeating gene expression patterns

**Fig. 1:**
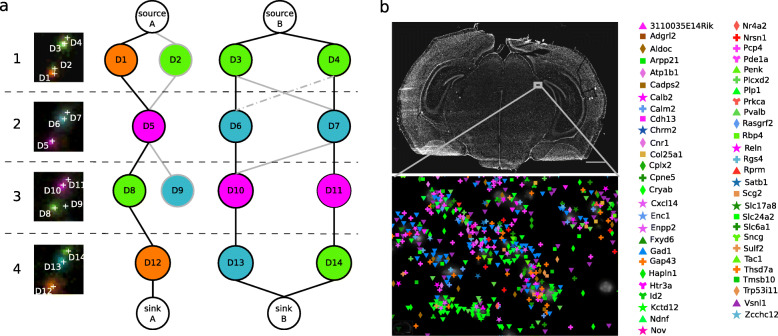
Graph-based ISS signal decoding and spatial mapping. **a** Cut-out composite images from four sequencing cycles (1–4) with four fluorescent channels (magenta, cyan, orange, and green representing the letters A, C, T, and G) are shown in the left panel. Each detected signal is marked with a white cross, labeled D1-D14, and represented as a node in the graphical model (with the same color and label). The graphical representation of the fluorescent signals results in two independent connected components, represented by two graphs A and B. Edges between nodes represent distance between signals, where bold lines represent direct connections (distance < *d*_th_), and dash-dotted lines represent forced connections (distance < *d*_max_). Solving the graph gives three black paths: {D1,D5,D8,D12}, {D3,D6,D10,D13}, {D4,D7,D11,D14}, corresponding to three decoded sequences: TAGT, GCAC and GCAG. **b** Overview of a full coronal section of a mouse brain with zoomed-in regions showing detected barcodes and corresponding genes. Scale bar, 1000 μm. Analyzed samples are available for interactive viewing at [17], and video tutorial on how to visualize spatial gene expression data is available at [18]

Our proposed ISS decoding of 5 sequencing cycles led to the detection of a total of approximately 0.18 to 2 M transcripts representing 82 to 97 different genes across all four brain sections. An example of achieved signal detection is shown in Fig. 1b, and all four brain sections are available for interactive viewing via TissUUmaps [19] at [17]. Instructions demonstrating how to use the viewer are available at [18]. Note that only a randomly selected fraction of the transcripts are shown at low resolution to optimize visualization interaction. Zooming in to a smaller part of the tissue will show all transcripts. Color coding, symbols, and their size can be modified to visualize single genes or combinations of genes, at multiple resolutions.

### ISS gene expression patterns show similarity with ISH-Allen Mouse Brain Atlas patterns

**Fig. 2:**
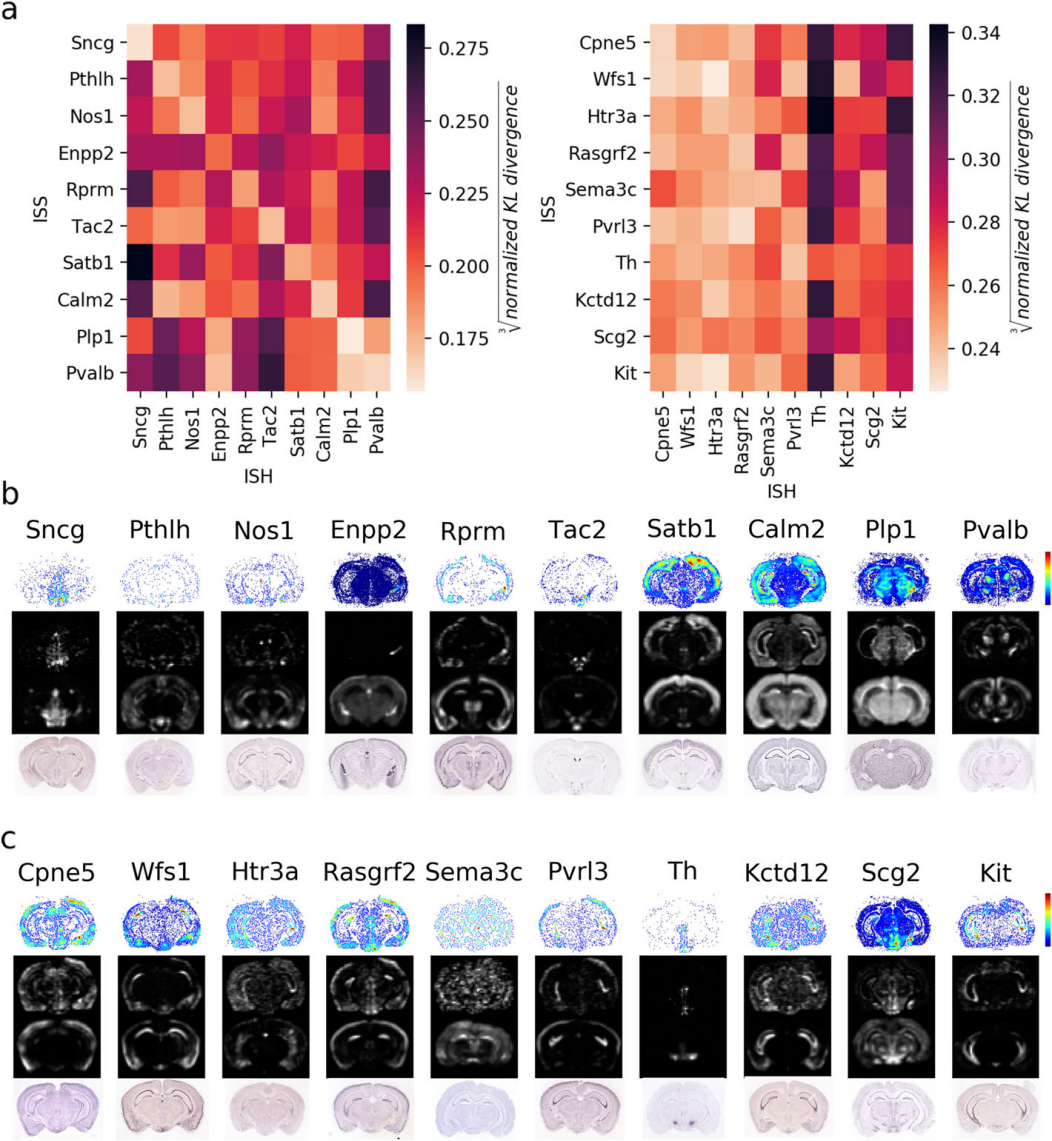
ISS patterns compared to the Allen Mouse Brain ISH-Atlas of one of the coronal mouse brain sections from mouse 1. **a** Detailed visualization of the 10 most (left) and least (right) matching genes as compared by KL divergence. **b** Visualization of spatial patterns of top-10 genes, from top to bottom: decoded ISS reads with density profile color code, low-resolution grayscale images of normalized ISS and Allen Brain Atlas ISH expression, and Allen Brain Atlas ISH data. **c** Visualization of spatial patterns of 10 least matching genes, from top to bottom as in **b**

Per-gene ISS gene expression patterns and ISH patterns from the Allen Mouse Brain Atlas were compared for the two hippocampal sections of mouse 1 using normalized Kullback-Leibler (KL) divergence. We sorted rows and columns of the KL divergence matrix by the difference between the KL divergence of a gene ISS pattern with its corresponding ISH pattern and the minimum KL divergence with the other genes. By doing so, the ISH patterns that best match a single ISS pattern (and vice versa) appear in the top-left quadrant of Fig. 2a (left), showing the top 10 matches. On the contrary, Fig. 2a (right) shows the result of the 10 least matching genes. All patterns are shown in Additional file 1: Fig. S1a. Corresponding patterns of the second hippocampal section are shown in Additional file 1: Fig. S1b and S2. Note that many of the poorly matching patterns may be due to the different modalities of the data, poor matching of brain slices, or even anatomical differences between the mice (n.b. ISS P25 mice are compared with ISH P56 mice).

### Color-space visualization of UMAP embedding reveals sharp edges and soft gradients in gene expression

**Fig. 3:**
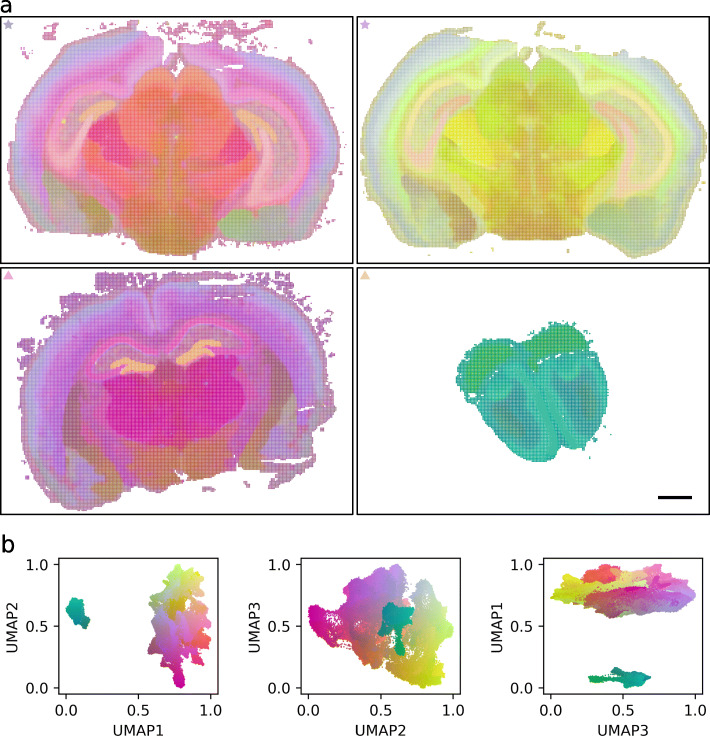
Color-space visualization of UMAP embedding. **a** Brain gene expression variations: each patch is color coded based on its gene expression profile projected in a 3D space. Patches with similar color have highly correlated gene expression profiles. Brain sections marked with a star in the top left corner are two consecutive sections of the same brain (mouse 1), while brain sections marked with a triangle are from a different mouse (mouse 2), one with location similar to the sections of mouse 1 and the other one from the olfactory region. Scale bar, 1 mm. **b** Visualization of the patch gene expression profiles in the dimensionality reduction space (three different projections of the same space), showing how the olfactory region (cyan) forms a cluster far away from the more centrally located slices

To visualize spatial variation in gene expression, each normalized spatial gene expression matrix was mapped into a 3-dimensional embedding using UMAP [15]. The axes of this new reference space were normalized to unit vectors and presented as an RGB color space, as shown in Fig. 3b for the full gene panel and in Additional file 1: Fig. S3b for the reduced gene panel. Each data point in the RGB space corresponds to a patch with a given 2D coordinate in the spatial brain map. Figure 3a (and Additional file 1: Fig. S3a) is thus the corresponding gene expression map, color coded based on the UMAP embedding. Note that patches with similar color have highly correlated gene expression profiles, while very different colors correspond to patches with larger differences in gene expression patterns. For example, the pyramidal cell layer in the hippocampus, as well as the dentate gyrus, form very distinct boundaries, while the layers of isocortex are more softly graded.

### Spatial gene expression clusters are highly reproducible across brains

**Fig. 4:**
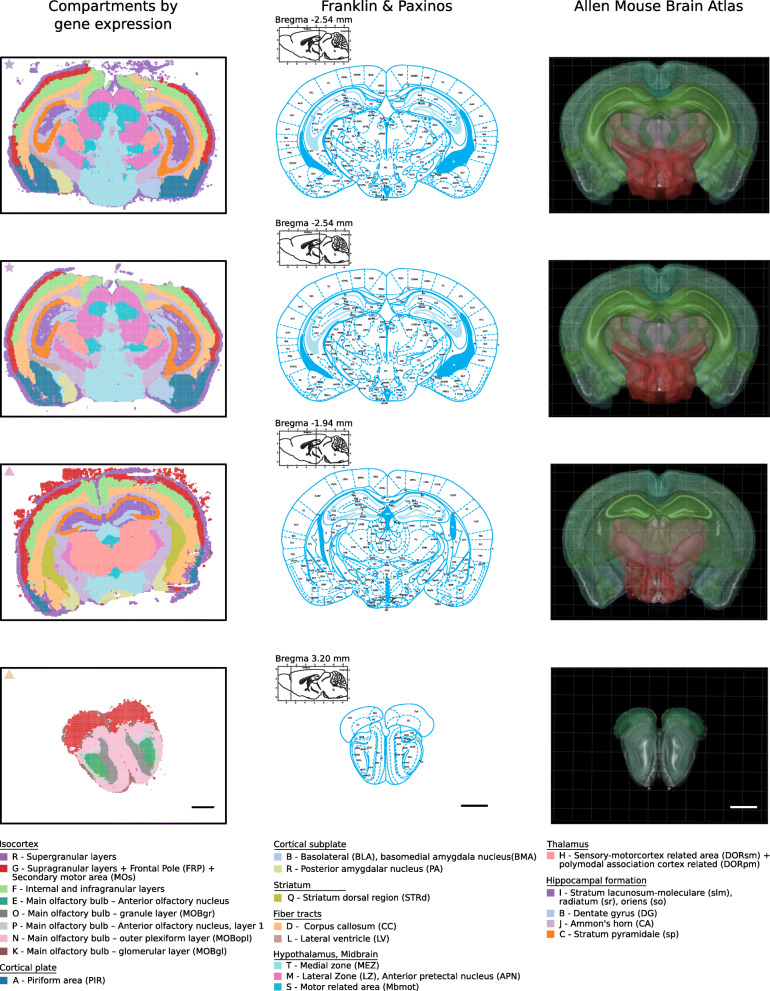
Spatial gene expression compartments are highly reproducible across brains. Visualization of 20 spatial compartments, with their annotations, defined by highly correlated clusters (right column). The annotations follow the Allen Mouse Brain Atlas (left column). For comparisons, we also provide the annotations by Franklin and Paxinos (central column). Note that brain sections are slightly angled medial-lateral as well as dorso-ventral. Sections from different mice are marked respectively with a star and a triangle in the top left corner. Scale bars, 1 mm

The continuous color space in Fig. 3 does not define distinct spatial compartments. A 50-dimensional UMAP representation of the data from the full gene panel was clustered using the Leiden clustering algorithm [20] resulting in the definition of 17 to 32 regions in the four different brain sections. Regions defined in the different brains were then matched based on the correlation of their gene expression profiles alone (Additional file 1: Fig. S4). The top 20 distinct spatial compartments in the cluster hierarchy are shown in Fig. 4 and their subclusters in Additional file 1: Fig. S5. All identified regions in the four brain sections can be iteratively visualized and explored in TissUUmaps at [17]. The top 20 distinct spatial compartments show high reproducibility between three sections containing the hippocampus, while the olfactory section has a distinctly different pattern. These spatial compartments show high correspondence with the landmarks in the Allen Mouse Brain Reference Atlas, and they can be readily annotated as shown in Fig. 4.

### Reproducible clustering is possible with as little as 18 genes

**Fig. 5:**
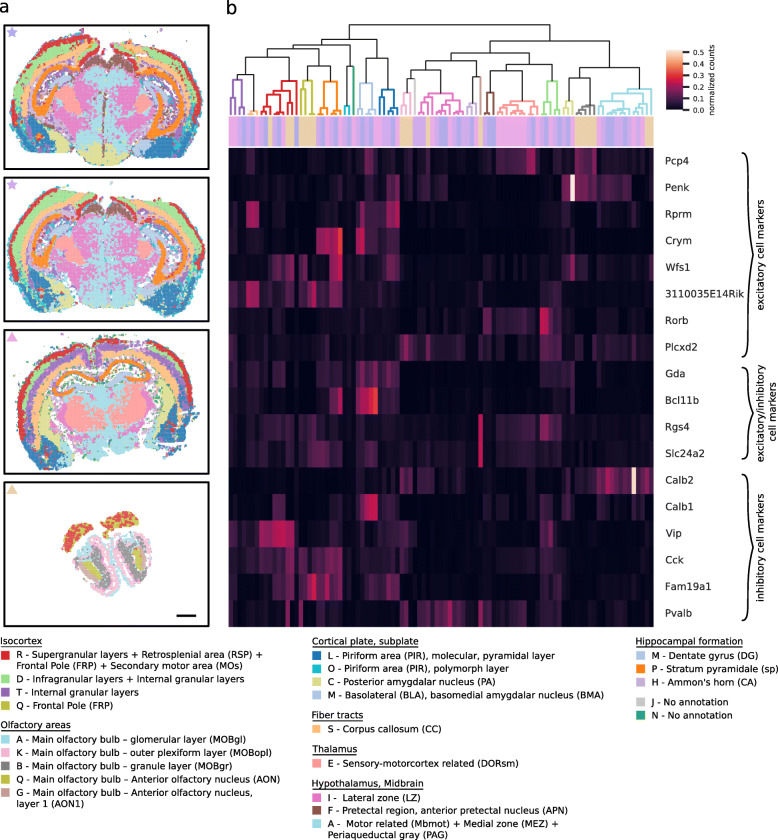
Reproducible definition of compartments is possible with as little as 18 genes. **a** Visualization of 20 compartments, with their annotations, defined by highly correlated clusters. Note that brain sections are slightly angled medial-lateral as well as dorso-ventral. Scale bar, 1 mm. **b** Hierarchical clustering of gene expression profiles based on correlation. Color coding according to regions listed at the bottom of the figure. Gene expression profiles for each cluster are defined by summing the expression of selected marker genes from all patches belonging to the cluster and normalizing by cluster size. Row colors represent sample id as defined by star and triangle markers in the top left corner for each brain section in **a**

Definition of spatial compartments in Fig. 4 relied on data from 78 genes. To explore the limitations of the approach, we successively reduced the gene set, down to a minimal set of 18 genes resulting in the definition of 19 to 28 clusters in the four brain sections. Clustering with the reduced gene set identified a lower number of regions for two of the four brain sections, while detecting a slightly higher number for the other two sections (respectively one and two additional clusters for the two brain section from mouse 2 in Fig. 5a) suggesting some overclustering due to the lower sequencing depth of the reduced gene panel dataset. We matched cluster gene expression profiles from the four brain sections based on correlation Fig. 5b, Additional file 1: Fig. S6, and show the top 20 spatial compartments in the cluster hierarchy in Fig. 5a and their subclusters in Additional file 1: Fig. S7. All regions identified with the reduced gene panel in the four brain sections can also be iteratively visualized and explored TissUUmaps at [17]. The identified spatial compartment based on a minimal set of 18 marker genes allows the possibility of unbiased differential expression analysis for the remaining 60 genes, as exemplified in Additional file 1: Fig. S8, S9.

### Cell morphology patterns vary between spatial compartments, but are not sufficient to define compartments

**Fig. 6:**
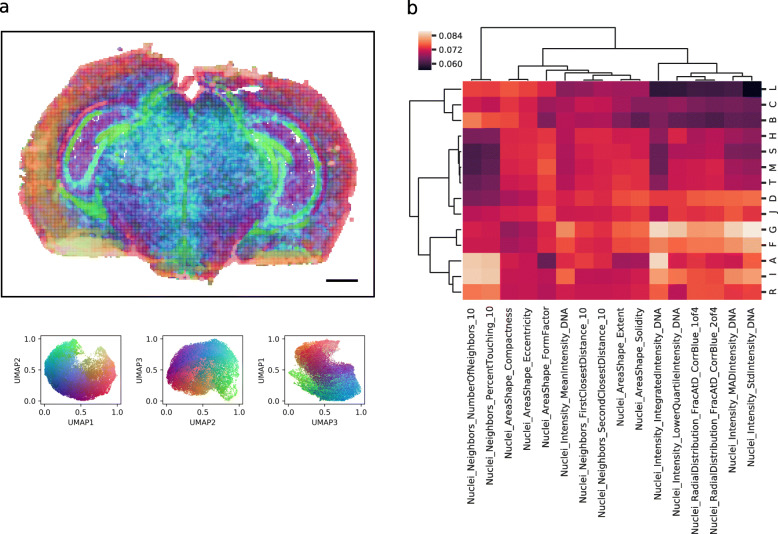
Spatial variation in cell morphology. **a** Visualization of morphological variation in one brain section using RGB color space shows some distinct compartments, while others cannot be differentiated. Scale bar, 1 mm. **b** Spatial compartments defined by gene expression (visualized in Fig. 4) are clustered based on cosine similarity in morphological profiles, where the profiles are defined by summing the normalized morphological features of all patches belonging to the compartment and scaling each feature by compartment size

We further explored clustering based on cell morphology and how local cell morphology correlates with the spatial compartments. Visualizing spatial variation in cell morphology in 3D color space (using the same approach as was applied for variations in gene expression), we can see that some distinct patterns appear (Fig. 6a), while noise levels are much higher than those for gene expression (shown in Fig. 3). Approaches to define more than a few clusters in this space were not successful due to the high noise levels. Next, we compared cell features within the spatial compartments defined by gene expression. While some spatial compartments show distinct morphological profiles (i.e., clusters B, C, and L corresponding to dentate gyrus, stratum pyramidale, and lateral ventricle sharing high density features, and clusters R, F, and G, corresponding to isocortex sharing high intensity features), others can be better identified based on their gene expression (i.e., hypothalamus and midbrain clusters T, M, and S of Fig. 4), since variations in cell morphology are very small (Fig. 6b).

### Spatial compartments are poorly defined by non-negative matrix factorization and SpatialDE analysis

Non-negative matrix factorization [13] provided a series of locally correlated gene expression maps representing distinct biological processes that show clear localized expression (Additional file 1: Fig. S10).

Also, SpatialDE [14] identifies spatially variable genes by clustering their spatial profiles based on a Gaussian-process-based prior. Genes with similar spatial variation are thus grouped together into spatially significant patterns. Initializing the number of patterns to 20, we obtain 15 and 13 spatial gene expression patterns defined by sets from 1 to 24 genes (Additional file 1: Fig. S11 and Additional file 1: Table S1). Although most of these patterns show clear spatial localization, they do not define spatial compartments, but rather represent high order biological processes resulting from correlated expression of specific gene expression patterns that encompass single or multiple compartments.

## Discussion

We analyzed ISS data of four mouse brain sections from two different individuals. Two of the four sections were provided by Qian et al. [21] and were processed by our graph-based decoding image analysis pipeline in order to extract the spatial gene expression. The study by Qian et al., although partially based on the same data, focuses on cell typing analyses of manually selected brain regions. Here instead, we show how the spatial gene expression can be used to segment spatial molecular compartments without the need of costly manual annotations.

Specifically, we have first decoded the spatial gene expression and related decoded expression patterns with ISH-Allen Mouse Brain Atlas patterns based on Kullback-Leibler divergence. We found that some gene expression patterns match strongly with the corresponding ISH patterns but others relate more poorly due to discrepancies in the cutting angles of tissue slices or due to different shape and size of samples. This highlights the challenge to map spatial gene expression data to a common reference in order to reliably define spatial anatomical compartments for further investigations. Many efforts are on the way to develop a common coordinate framework for cell atlases, such as the Human Cell Atlas [22], the Human BioMolecular Atlas Program [23], and the Lifetime FET flagship consortium [24]. The method presented here will support these efforts by providing a solution for an automated gene-driven segmentation of tissue regions irrespective of translation and rotation of the tissue sections. This could be extremely valuable for human tissue sections (both surgically removed or post-mortem) that due to their larger size are sometimes cut in smaller pieces and where the exact location of the biological sample is often unknown.

Here, we showed that mapping the spatial gene expression to a common RGB color space can reveal both sharp and soft transition of expression across the samples. Moreover, cluster analysis of the spatial expression profiles using as few as 18 marker genes can reliably identify visually consistent spatial compartments. It should be noted here that our method can also be applied to data from other spatially resolved methods, such as spatial transcriptomics [25]. While ISS is a targeted approach and offers single cell resolution, spatial transcriptomics is untargeted and limited by the spot size and distance between spots. However, with deeper sequencing, spatial transcriptomics may be able to find smaller differences in expression between neighboring regions (but with lower spatial resolution). Ortiz et al. [25] have used 7663 genes and a reduced palette of 266 genes to define 181 molecular clusters within the mouse brain. Still, not all annotated regions, such as provided by Paxinos and Franklin [26], can easily be separated, e.g., separating the primary visual cortex from secondary visual cortex or auditory cortex is challenging. Here, we focused on 18 marker genes that can solely separate distinct brain regions, but more (carefully selected) marker genes may be needed to achieve a finer resolution of brain regions.

We also investigated if spatial compartments can be accurately defined only based on cellular morphological features. We showed that morphological variation does not always follow gene expression, and different spatial compartments can be defined by various cell types with common morphological features but distinct gene expression profiles. It is yet to be explored if combining gene expression and morphological information can improve compartment definition. Similarly, in the field of cell typing classification schemes and nomenclatures based on gene expression are advantageous over providing classifications purely based on morphological or physiological criteria because of their richness of data [27]. Such transcriptomic nomenclatures can be combined with additional features but currently serve as the basis for cell type classifications.

## Conclusion

The molecular architecture of cells in the brain is very diverse and the brain as such is a very heterogeneous organ. Technologies, like single-cell RNA sequencing or in situ sequencing, offer high-throughput measurements of RNA and allow to capture the molecular diversity within tissue sections. Here, we used an approach that relies on gene expression data to segment and identify compartments in the brain. We believe that the identification of brain compartments based on gene expression features has more discriminative power than morphological feature analysis and thus can better guide the fully automated identification of spatial compartments for further investigations in relation to development or disease.

## Methods

### In situ sequencing (ISS) data generation

The design and experimental details of the ISS assay used to produce the data for this paper is described in detail in Qian et al. [21]. The raw images of two brain sections (mouse 1, brain sections marked with a star in all figures) were provided by Qian et al. The other two brain sections belonging to a different mouse (mouse 2, brain sections marked with a triangle in all figures) were probed independently in a different set of experiments but with the same experimental conditions. In brief, 10 μm fresh frozen brain tissues from CD1 male mice (postnatal day 25) were probed with 95 nucleotide (nt) long padlock probes. Padlock probes contained a 4 nt sequencing by ligation barcode and a 20 nt sequencing by hybridization barcode (called “base 5”). A 97-gene panel and a 82-gene overlapping panel (Additional file 2: Table S2) were probed in two different experiments and imaged on an epifluorescence microscope AxioImager.Z2 (Zeiss) with × 20/0.8 objective. At each sequencing cycle, the tissue sample was stained and imaged in six fluorescent channels: a nuclei channel, a general stain channel (used as reference channel for each barcode probe), and four color channels; one for each letter of the genetic code (A, C, G, T), following the protocol summarized by Hilscher et al. [28]. The result of the experimental procedure was image data with three spatial dimensions (x, y, z), one color dimension encoding the different fluorescent channels, and a temporal dimension for the different imaging/sequencing cycles. Below, we describe the analysis pipeline. The output is the spatial coordinates of decoded barcodes along with scores to assess the quality of the decoding.

### Gene selection

The gene panels for the ISS experiments were selected based on single-cell RNA sequencing data from the hippocampus (roughly 28,000 genes [29]) and using pci-Seq trimmed to two subsets of 82 and 97 genes, respectively [21]. A total number of 78 genes passed quality filtering for our clustering approach. The expression of the 78 marker genes was not limited to the hippocampus: excitatory and inhibitory cell type markers are often shared between brain regions. For our “reduced” dataset, we aimed for a limited set of genes specific for different cell types. We settled for 18 marker genes—6 inhibitory markers (Pvalb, Vip, Cck, Fam19a1, Calb1, Calb2), 6 Pan-excitatory markers (Rprm, Crym, Wfs1, Pcp4, Plcxd2, 3110035E14Rik), 2 markers associated to neocortical layers (Rorb—Layer 4, Penk—Layer 6), and 4 markers that are shared between excitatory and inhibitory cells (Slc24a2, Bcl11b, Gda, Rgs4) [30]. A smaller number of genes led to less robust region definitions, and while a larger number of genes resulted in more distinctly defined regions, we found this set of 18 genes to be a good balance between data reduction and region definition.

### Image registration, tiling, and normalization

Image maximum intensity projections were aligned to compensate for chromatic aberration of fluorescent channels and for misalignments among successive imaging cycles caused by repetitive washing and staining procedures. Each color channel was aligned to the general stain channel of the respective sequencing cycle, applying a translation to compensate for chromatic aberration. Successively, general stain channel images from different sequencing cycles were rigidly aligned to a common reference sequencing cycle using multiresolution image registration [31]. The same transformations were then applied to the related color channels in order to create a common coordinate space. For each registration, a transformation matrix was estimated using normalized cross-correlation metric optimized with Adaptive Stochastic Gradient Descent [31]. After registration of the whole slide images, each image was tiled in smaller non-overlapping patches of 1028 × 1028 pixel size in order to split the dataset in smaller multidimensional tensors used for parallelization of later operations and optimize memory resources. A second alignment step was then performed for each individual tensor to locally align channels and cycles repeating the procedure of the first alignment.

Images were normalized by scaling the intensity values between the background intensity and the signal intensity estimated from *n* random 128 × 128 pixel patches from the whole slide image of the respective channel and cycle. The background intensity was defined as the mean intensity of the patch modes, and the signal intensity was defined as the 99th percentile of 98th patch percentiles.

### Signal candidate detection, merging, and probability prediction

Signal candidates were extracted with an h-maxima transform [32] from the normalized images after a top-hat filtering used for enhancing bright spots and attenuate background. Therefore, all local maxima with an h-maxima greater than a given threshold *h* are considered as signal candidates.

Due to broad emission spectra and imperfect washing procedures, fluorescent signals can bleed-through to adjacent channels or sequencing cycles and cause multiple false detections of the same signal. As a barcode should only represent a single letter at a given sequencing cycle, each barcode should only fluoresce in a single color channel (other than the general stain) per sequencing cycle. Therefore, signal candidates of a given sequencing cycle are grouped across color channels such that overlapping detection or detections that are adjacent in a four-connectivity pixel grid are merged together, keeping the signal candidate in the channel with highest intensity.

Many of the signals detected by the h-maxima transform are likely to be noise, also after the merging step. Therefore, a signal probability prediction describing the probability of a signal candidate of being signal or noise was calculated. This was done using a convolution neural network (CNN, Additional file 1: Fig. S12), implemented and trained in-house on a subset of manually annotated candidate signals (nominated by the h-maxima transform) from multiple ISS experiments. Using 5 × 5 pixel windows centered in each signal candidate (selected randomly across all color channels and sequencing cycles) as training data, the CNN learned the underlying discriminative features to predict the similarity between a signal candidate and a true signal.

### Graph-based signal decoding

Finally, signal candidates and probability predictions were combined in a graphical model as follows to resolve and decode the gene sequences across fluorescent channels and sequencing cycles: Signal candidate detections are represented in the graph as *D* nodes (colored nodes in Fig. 1a). Each *D* node consists of a pair of nodes connected by an edge with weight *w*_*i*_ equal to:

$$ {w}_i=-\log \left({p}_i\right), $$

where *p*_*i*_ is the probability prediction of the signal candidate detection *i*. Relationships among signal candidate detections belonging to different sequencing cycles are encoded as edges connecting *D* nodes (Fig. 1a). Each edge connecting a pair of *D* nodes has a weight proportional to the Euclidean distance between the signal candidate detections represented, specifically:

$$ {w}_{ij}=-\log \left(\frac{1}{1+k{d}_{ij}}\right), $$

where *d*_*ij*_ is the Euclidean distance between detection *i* and *j*, and *k* is a weighting parameter used to modulate the contribution of *d*_*ij*_. In order to build the graph, signal candidate detections are searched for connected components between sequencing cycles within a maximum connection distance *d*_*th*_. Each connected component can be represented as a graph with *D* nodes encoding candidate detections and edges representing connections. Each of the connected components that are found is then refined by adding edges between not connected *D* nodes belonging to consecutive sequencing cycles that are closer than a maximum distance *d*_max_. Nodes of the first and last sequencing cycles are then connected respectively to a source and a sink node. Finally, connections are removed between detections not belonging to consecutive sequencing cycles and the graph is solved by maximum flow of minimum costs between the source and the sink [33].

### Quality of decoded barcodes

A quality metric *Q*_*s*_ was assessed for each decoded sequence *s*, encoded by the set of detections *D*_*sb*_ : *b* ∈ [1, *n*], where *n* is the number of sequencing cycles. The quality score per sequence is proportional to the probability predictions, intensities, and distances of the signal candidate detections that form the sequence and is defined as:

$$ {Q}_s={\mu}_s\cdot {\sum}_{b=1}^n{Q}_{sb}, $$

where *Q*_*sb*_ is the quality score of each decoded base that forms the sequence and *μ*_*s*_ is a function proportional to the maximum distance between the detections that form the sequence. Specifically, *Q*_*sb*_ is defined as:

- If multiple detections {*D*_*sb*1_, …, *D*_*sbk*_}, with a probability prediction higher than 0.5, other than *D*_*sb*_ were detected and merged in a given cycle:
  $$ {Q}_{sb}=\frac{I_{D_{sb}}{p}_{D_{sb}}}{I_{D_{sb}}{p}_{D_{sb}}+\max \left({I}_{D_{sb1}}{p}_{D_{sb1}},\dots, {I}_{D_{sb k}}{p}_{D_{sb k}}\right)}, $$
- Otherwise,
  $$ {Q}_{sb}={p}_{D_{sb}} $$

where *I* and *p* are respectively intensity value and probability prediction of a given candidate. In order to penalize sequences whose detections are far apart from each other, *μ*_*s*_ is defined as:

$$ {\mu}_s=1-\frac{\log \left(1+d\right)}{\sigma }, $$

and clipped between 0 and 1 values, where *d* is the maximum distance between signal candidate detections composing a sequence and *σ* is a parameter weighting the penalty. Parameter *σ* is empirically set to the value that maximizes the area under the receiver operating characteristics from the true positive rate and false positive rate evaluation based on sequences of targeted barcodes.

### Evaluation of gene expression patterns in relation to the Allen Mouse Brain Atlas

We verified the output of the decoded gene expression patterns from two mouse brain sections with respect to 3D grid expression data from the in situ hybridization (ISH) Allen Mouse Brain Atlas [1]. The Allen Mouse Brain Atlas provides genome-wide in situ hybridization data for approximately 20,000 genes processed with a data processing pipeline for extracting 3D grid gene expression data [34, 35]. The output of the data processing is a 67 × 41 × 58 voxel grid with quantified gene expression values for each gene. For each of the gene expression patterns decoded in the two brain sections, we computed the KL divergence with respect to grid expression patterns from the ISH atlas in order to assess spatial pattern similarities. Gene expression patterns from ISS were first scaled to match the resolution of the atlas voxel grid. Second, a probability density function was estimated for each gene using a Gaussian kernel with covariance factor of 0.05. Both ISS and ISH gene expression patterns were then normalized such that the total mass of each probability density function became equal to 1. We then selected the two (out of 67) coronal levels that best match the ISS sections for computing the Kullback-Leibler divergence between normalized expression patterns. The coronal level showing the closest gene-gene similarity was further selected for visualization purposes.

### Gene expression matrix generation and UMAP embedding

Prior to investigating spatial gene expression variations, the output from the sequence decoding step was filtered independently for each brain section by first removing low quality reads applying a quality threshold of 2, and excluding low-expressed genes that had a total count < 500. Successively, a gene expression matrix was constructed for each sample where rows represent overlapping patches of the tissue sample and columns represent targeted genes. Gene expression matrices were generated using square tiles of size 128 × 128 pixels and overlap of 128 pixels, so that each patch represents genes from a region of size of 384 × 384 pixels (~ 125 μm^2^). Each entry in the matrix represents the expression level of a particular gene in a given patch (i.e., total count of reads decoded inside the patch). An additional filtering step excludes patches with less than 10 reads from further evaluations. Thus, a total of 78 genes from all samples passed the filtering steps, resulting in a total of 4 genes excluded from the analysis (i.e., Chodl, Cort, Crh, Pax6). The expression table of each sample was thereafter normalized, stabilizing variance using Anscombe’s transform and successively regressing out the logarithmic total counts of reads per patch in order to exclude unwanted sources of variation such as number and size of cells in each patch. Next, the expression matrices were scaled feature-wise to zero mean and unit variance. Each filtered and normalized expression matrix was thereafter mapped into a common 3-dimensional embedding using UMAP [15]. The axes of the 3-dimensional embedding were normalized to unit vectors and coordinates of each projected patch in this new reference space were used to map each patch in the RGB color space.

### Spatial gene expression clusters and differential gene expression analysis

We then investigated if the gene expression from the four sections clusters in common brain compartments. The gene expression matrices generated in the previous analysis were mapped together into a 50-dimensional space using UMAP and clustered using the Leiden clustering algorithm [20]. Gene expression profiles of identified clusters were then normalized by cluster area and total gene counts, and grouped together with correlation-based hierarchical clustering.

### Clustering with a minimal gene set

In order to perform differential expression analysis between spatial compartments without bias introduced by the clustering itself, we also define compartments by clustering the gene expression of a limited set of 18 marker genes. Marker genes were chosen hierarchically from scRNA literature according to strongly expressed genes for interneurons, pyramidal cells, and non-neuronal cells (the “Gene selection” section). Again, dimensionality of the marker gene expression profiles of the two brains was reduced in a common 50-dimensional space. Next, patches of each brain section were clustered individually with the Leiden clustering algorithm [20]. To verify region similarity, differential expression analysis based on Wilcoxon rank-sum statistical test implemented in Seurat [36] was applied to the genes excluded from the reduced gene panel between and within the defined compartments of two brain sections.

### Morphological feature analysis

We then conducted a morphological feature analysis to study how cell morphology relates with gene expression profiles. We first segmented cell nuclei from the respective channel of one brain section with CellProfiler [37] and successively extracted 16 morphological features using CellProfiler Analyst [16]. We removed border artifacts from the single tiles before normalizing morphological features between bounds that visually highlight spatial profiles. We further removed outliers based on nuclei area and shape caused by segmentation errors. This resulted in a feature vector of length 16 for each segmented cell. Using the same patches defined before for spatial gene expression analysis, we created a matrix where each entry represents local cell morphology. Next, we kept only patches that contained more than three cells and regressed out the total number of cells per patch. We then visualized spatial variation in cell morphology (mapping the extracted patch morphological profiles in RGB color space as done for visualizing spatial gene expression variation) and compared morphological profiles of spatial compartments defined through gene expression clustering for relating gene expression with cell morphology.

### Non-negative matrix factorization and SpatialDE analysis

Non-negative matrix factorization is well suited for its non-negativity feature to biological contexts where biological signals can be naturally present or absent. We therefore translated normalized expression values into a positive space for applying non-negative matrix factorization analysis [13]. The expression matrix *E* is thus factorized into two nonnegative matrices *W* and *H* initializing the number of factors to 20. An approximation of the gene expression matrix *E* is then reconstructed as a linear combination of column feature vectors in *H* weighted by the contribution of each gene column in matrix *W*:

$$ E= WH $$

Columns feature vectors of matrix *H* represent therefore co-expression patterns (also called metagenes) shared in the two brains, often corresponding to real biological patterns. And rows of matrix *W* represent the contribution of each gene in each metagene.

We also investigated gene expression variation in tissue samples with SpatialDE [14], a framework based on Gaussian process regression that identifies genes with spatially significant gene expression patterns. We ran SpatialDE analysis on normalized gene expression matrices generated from square patches of 512 px size and 64 px overlap, due to a higher demand of computational resources by the tool. SpatialDE classified all targeted genes as significantly spatially varying as was expected due to the choice of gene panel (Additional file 1: Fig. S11a). We then performed SpatialDE “automatic expression histology” analysis consisting in a spatial clustering of similarly spatially varying expression patterns based Gaussian Mixture Models with a spatial Gaussian prior on the cluster centroids.

## Supplementary information

**Additional file 1: Fig. S1.** Allen Mouse Brain ISH-Atlas comparison. **Fig. S2.** ISS patterns of the coronal mouse brain section from Fig. S1b compared to the Allen Mouse Brain ISH-Atlas. **Fig. S3.** Color-space visualization of UMAP embedding for the reduced gene panel expression (18 markers). **Fig. S4.** Hierarchicalclustering of the identified cluster gene expression profiles using the full gene panel. **Fig. S5.** Visualization of the identified sub-clusters of the 20 spatial compartments displayed in Fig.4 for the four brain sections. **Fig. S6.** Hierarchical clustering of the identified cluster gene expression profiles using the reduced gene panel (18 genes). **Fig. S7.** Visualization of theidentified sub-clusters of the 20 spatial compartments displayed in Fig. 5 for the four brain sections. **Fig. S8.** Differential expression analysis of identified brain compartments in two brainsections (mouse 1). **Fig. S9.** Cluster gene expression profiles of top five differentially expressed genes in each identified spatial compartment between two brain sections (mouse 1). **Fig.S10.** Non-Negative Matrix Factorization Analysis. **Fig. S11.** SpatialDE analysis for two of the brain sections. **Fig. S12.** Convolutional Neural Network Architecture for signal candidatepredictions. **Table S1.** a,b Spatial patterns composition of SpatialDE “Automatic Expression Histology”.

**Additional file 2: Table S2.** ISS gene panel and probe design.

## Data Availability

All software was developed in Python 3 using open source libraries, and data processing of pipeline workflows was carried out using Anduril2 analysis framework [38]. The processing pipelines, data, and the software version used to generate the analysis results and figures presented in this paper are available at (10.5281/zenodo.3928219) [39] or from our github repository https://github.com/wahlby-lab/graph-iss.
