# Supplementary material for: Automated identification of the mouse brain’s spatial compartments from in situ sequencing data
Source: BMC Biol. 2020 Oct 19;18:144. doi: 10.1186/s12915-020-00874-5 (PMC7574211; doi:10.1186/s12915-020-00874-5)
Supplement: Supplementary file 1 — Additional file 1: Fig. S1. Allen Mouse Brain ISH-Atlas comparison. Fig. S2. ISS patterns of the coronal mouse brain section from Fig. S1b compared to the Allen Mouse Brain ISH-Atlas. Fig. S3. Color-space visualization of UMAP embedding for the reduced gene panel expression (18 markers). Fig. S4. Hierarchicalclustering of the identified cluster gene expression profiles using the full gene panel. Fig. S5. Visualization of the identified sub-clusters of the 20 spatial compartments displayed in Fig.4 for the four brain sections. Fig. S6. Hierarchical clustering of the identified cluster gene expression profiles using the reduced gene panel (18 genes). Fig. S7. Visualization of theidentified sub-clusters of the 20 spatial compartments displayed in Fig. 5 for the four brain sections. Fig. S8. Differential expression analysis of identified brain compartments in two brainsections (mouse 1). Fig. S9. Cluster gene expression profiles of top five differentially expressed genes in each identified spatial compartment between two brain sections (mouse 1). Fig.S10. Non-Negative Matrix Factorization Analysis. Fig. S11. SpatialDE analysis for two of the brain sections. Fig. S12. Convolutional Neural Network Architecture for signal candidatepredictions. Table S1. a,b Spatial patterns composition of SpatialDE “Automatic Expression Histology”. [file 12915_2020_874_MOESM1_ESM.pdf]

Figure S1. Allen Mouse Brain ISH-Atlas comparison. **a,b)** Visualization of normalized KL divergence between in situ sequencing (ISS) spatial gene expression patterns and in situ hybridization patterns from Allen Mouse Brain Atlas for two brain sections (mouse 1) . Rows and columns are sorted by the difference between the KL divergence of a gene ISS pattern with its corresponding ISH pattern and the minimum KL divergence with the other genes. Such that, patterns that uniquely match their pair appear in the top-left quadrant.

Figure S2. ISS patterns of the coronal mouse brain section from Fig. S1b compared to the Allen Mouse Brain ISH-Atlas. **a)** Detailed visualization of the 10 most (left) and least (right) matching genes as compared by KL divergence. **b)** Visualization of spatial patterns of top-10 genes, from top to bottom: decoded ISS reads with density profile color code, low resolution gray scale images of normalized ISS and Allen Brain Atlas ISH expression, and Allen Brain Atlas ISH data. **c)** Visualization of spatial patterns of 10 least matching genes, from top to bottom as in b.

Figure S3. Color-space visualization of UMAP embedding for the reduced gene panel expression (18 markers). **a)** Brain gene expression variations: each patch is color coded based on its gene expression profile projected in a 3D space. Patches with similar color have highly correlated gene expression profiles. Brain sections with different marker symbols are from different individuals. Scale bar: 1 mm. **b)** Visualization of the patch gene expression profiles in the dimensionality reduction space (three different projections of the same space). Note that the two brain sections of mouse 1 (star marker) are not shifted in the color space as in Figure 3, suggesting that the selected genes of the reduced gene panel are less affected by technical batch effect than others.

Figure S4. Hierarchical clustering based on correlation of the identified cluster gene expression profiles using the full gene panel. Clusters belonging to the spatial compartments shown in Figure 4 are here represented with the same color code.

Figure S5. Visualisation of the identified sub-clusters of the 20 spatial compartmentents displayed in Figure 4 for the four brain sections. Each row represents a subdivision of spatial compartments shown in Figure 4 with the same color code.

Figure S6. Hierarchical clustering based on correlation of the identified cluster gene expression profiles using the reduced gene panel (18 genes).

Figure S7. Visualisation of the identified sub-clusters of the 20 spatial compartmentents displayed in Figure 5 for the four brain sections. Each row represents a subdivision of spatial compartments shown in Figure 5 with the same color code.

Figure S8. Differential expression analysis of identified brain compartments in two brain sections (mouse 1) with an internal average correlation >0.8. Combined normalized gene expression profiles for each expressed gene excluded from the reduced gene panel.

Figure S9. Cluster gene expression profiles of top five differentially expressed genes in each identified spatial compartment (with an internal average correlation >0.8) between two brain sections (mouse 1). Violin plots of normalized expression are shown for the top 5 genes based on average fold change with adjusted p-values <0.01 (Wilcoxon Rank Sum statistical test).

Figure S10. Non-Negative Matrix Factorization Analysis. **a,b)** Visualization of non-negative matrix factorization single components that represent co-expression patterns of two of the brain sections. **c)** Visualization of the weight matrix representing the contribution of each gene to the different co-expression patterns.

Figure S11. SpatialDE analysis for two of the brain sections. **a)** Fraction of variance explained by spatial variation (FSV) versus significance of spatial variation for all targeted genes. **b,c)** Visualization of SpatialDE “Automatic Expression Histology” analysis. Colorbars represent expression levels of each spatial pattern. The number of genes contributing to the pattern are listed in the title of each figure, and detailed in Table S1a,b.

Figure S12. Convolutional Neural Network Architecture for signal candidate predictions. The network takes as input a 5x5 pixel window centred in each signal candidate detection and provides probability predictions for the candidate to be signal and noise.

Table S1a. Spatial patterns composition of SpatialDE “Automatic Expression Histology” (displayed in Figure S11b).

Table S1b. Spatial patterns composition of SpatialDE “Automatic Expression Histology” (displayed in Figure S11c).

Figure S1

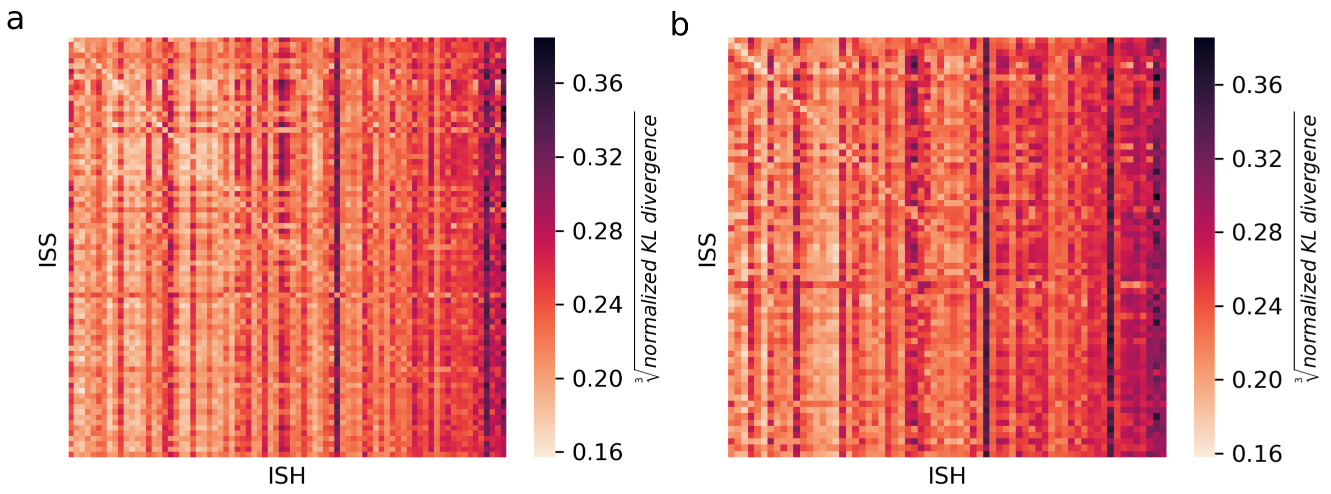
a
ISS
ISH
0.36
0.32
0.28
0.24
0.20
0.16
∛normalized KL divergence
b
ISS
ISH
0.36
0.32
0.28
0.24
0.20
0.16
∛normalized KL divergence

Figure S2

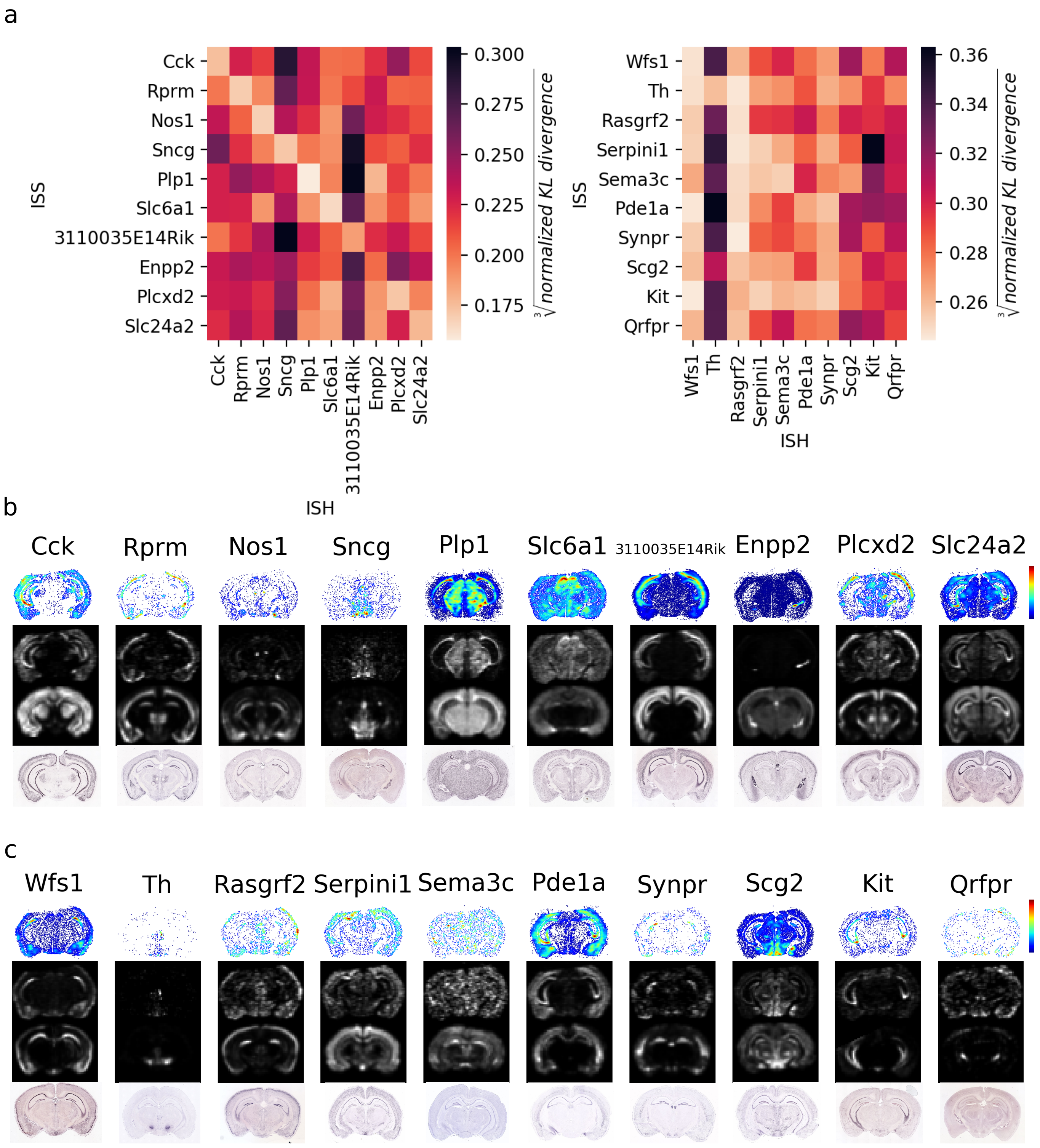
a
Cck
Rprm
Nos1
Sncg
Plp1
Slc6a1
3110035E14Rik
Enpp2
Plcxd2
Slc24a2
ISS
ISH
0.300
0.275
0.250
0.225
0.200
0.175
∛normalized KL divergence
Wfs1
Th
Rasgrf2
Serpini1
Sema3c
Pde1a
Synpr
Scg2
Kit
Qrfpr
0.36
0.34
0.32
0.30
0.28
0.26
b
c

Figure S3

a

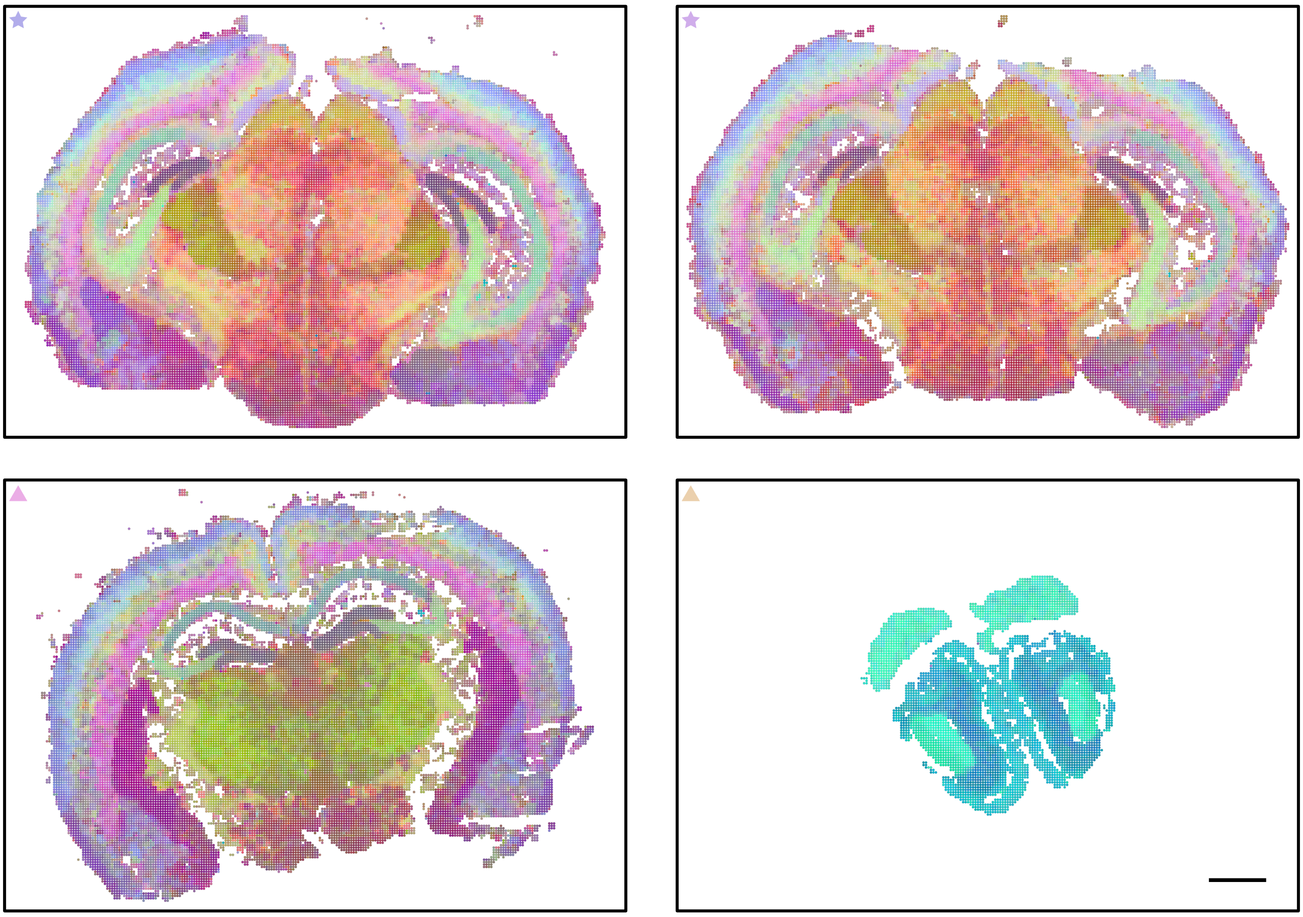

b

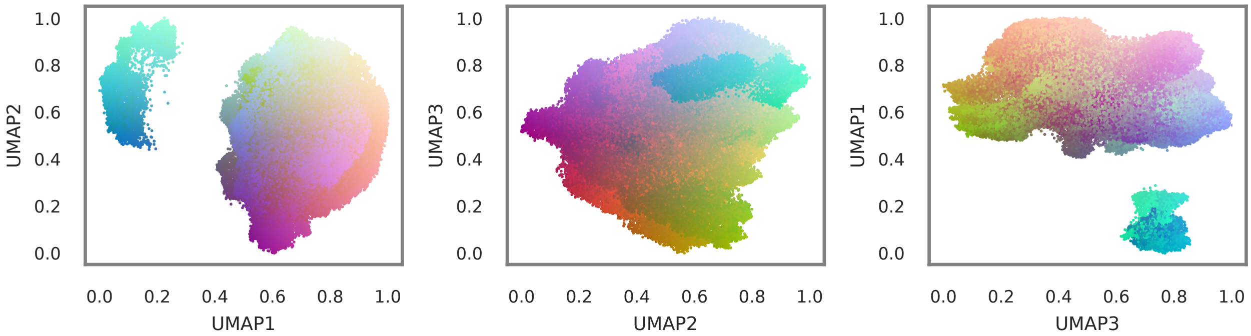

Figure S4

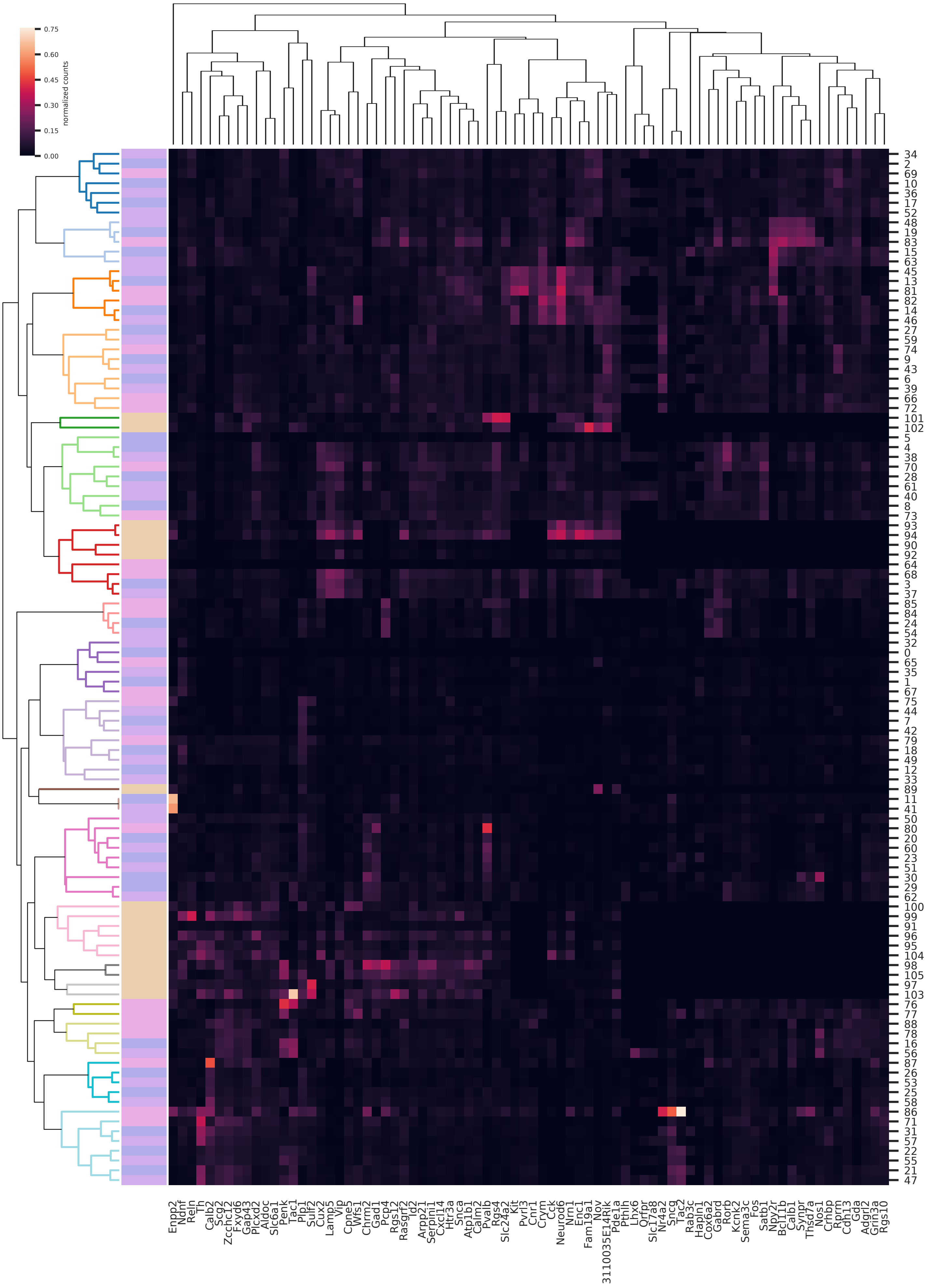
normalized counts
0.75
0.60
0.45
0.30
0.15
0.00
Enpp2 Ndnf Reln Th Calb2 Scg2 Zcchc12 Fxyd6 Gap43 Plcxd2 Aldoc Slc6a1 Penk Tac1 Plp1 Sulf2 Cux2 Lamp5 Vip Cpne5 Wfs1 Chrm2 Gad1 Pcp4 Rgs12 Rasgrf2 Id2 Arpp21 Serpini1 Cxcl14 Htr3a Snca Atp1b1 Calm2 Pvalb Rgs4 Slc24a2 Kit Pvrl3 Cnr1 Crym Cck Neurod6 Nrn1 Enc1 Fam19a1 Nov 3110035E14Rik Pde1a Pthlh Lhx6 Qrfpr Slc17a8 Nr4a2 Sncg Tac2 Rab3c Hapln1 Cox6a2 Gabrd Rorb Kcnk2 Sema3c Fos Satb1 Npy2r Bcl11b Calb1 Synpr Thsd7a Nos1 Crhbp Rprm Cdh13 Gda Adgrl2 Grin3a Rgs10
34 2 69 10 36 17 52 48 19 83 15 63 45 13 81 82 14 46 27 59 74 9 43 6 39 66 72 101 102 5 4 38 70 28 61 40 8 73 93 94 90 92 64 68 3 37 85 84 24 54 32 0 65 35 1 67 75 44 7 42 79 18 49 12 33 89 11 41 50 80 20 60 23 51 30 29 62 100 99 91 96 95 104 98 105 97 103 76 77 88 78 16 56 87 26 53 25 58 86 71 31 57 22 55 21 47

Figure S5

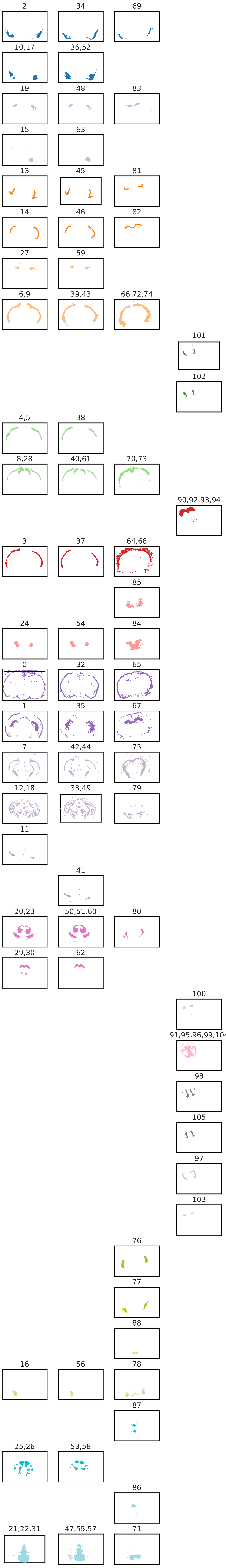

2
34
69
10,17
36,52
19
48
83
15
63
13
45
81
14
46
82
27
59
6,9
39,43
66,72,74
101
102
4,5
38
8,28
40,61
70,73
90,92,93,94
3
37
64,68
85
24
54
84
0
32
65
1
35
67
7
42,44
75
12,18
33,49
79
11
41
20,23
50,51,60
80
29,30
62
100
91,95,96,99,104
98
105
97
103
76
77
88
16
56
78
87
25,26
53,58
86
21,22,31
47,55,57
71

Figure S6

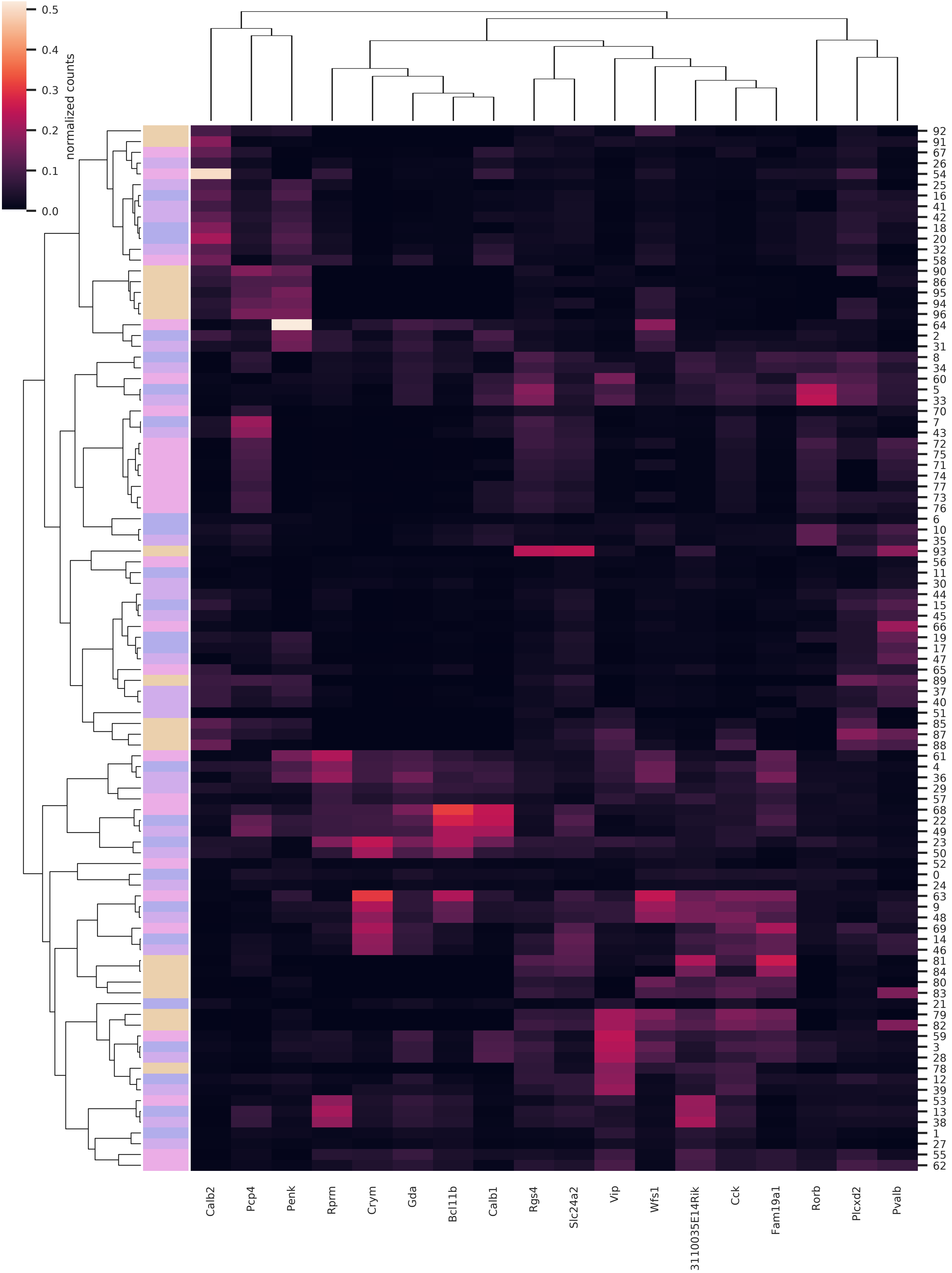
0.5
0.4
0.3
0.2
0.1
0.0
normalized counts
Calb2
Pcp4
Penk
Rprm
Crym
Gda
Bcl11b
Calb1
Rgs4
Slc24a2
Vip
Wfs1
3110035E14Rik
Cck
Fam19a1
Rorb
Plcxd2
Pvalb
92
91
67
26
54
25
16
41
42
18
20
32
58
90
86
95
94
96
64
2
31
8
34
60
5
33
70
7
43
72
75
71
74
77
73
76
6
10
35
93
56
11
30
44
15
45
66
19
17
47
65
89
37
40
51
85
87
88
61
4
36
29
57
68
22
49
23
50
52
0
24
63
9
48
69
14
46
81
84
80
83
21
79
82
59
3
28
78
12
39
53
13
38
1
27
55
62

Figure S7

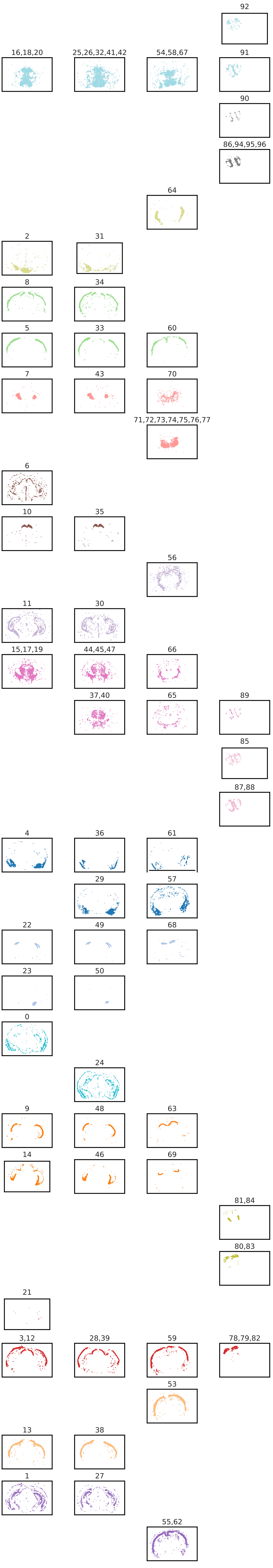
92
16,18,20
25,26,32,41,42
54,58,67
91
90
86,94,95,96
64
2
31
8
34
5
33
60
7
43
70
71,72,73,74,75,76,77
6
10
35
56
11
30
15,17,19
44,45,47
66
37,40
65
89
85
87,88
4
36
61
29
57
22
49
68
23
50
0
24
9
48
63
14
46
69
81,84
80,83
21
3,12
28,39
59
78,79,82
53
13
38
1
27
55,62

Figure S8

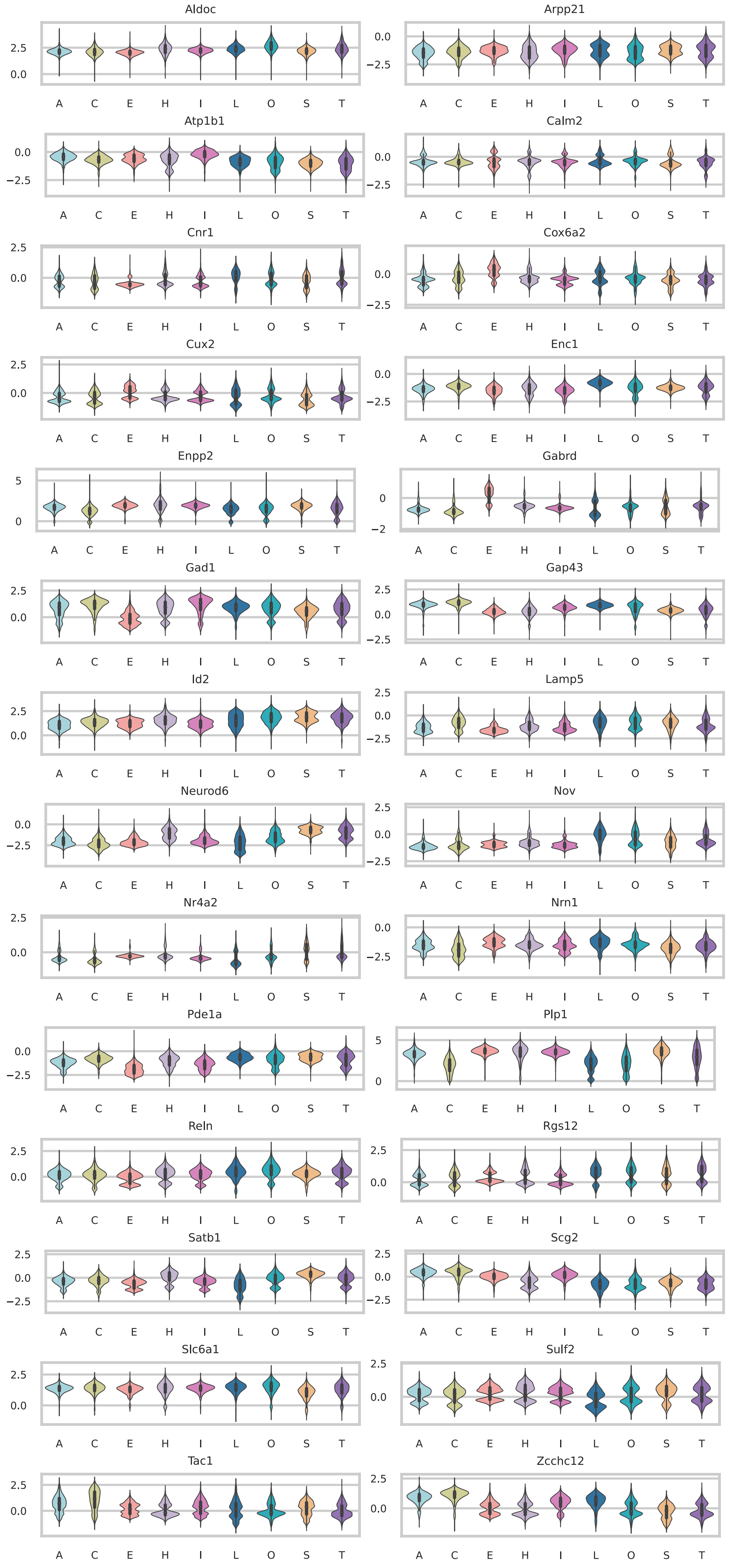
Aldoc
Arpp21
Atp1b1
Calm2
Cnr1
Cox6a2
Cux2
Enc1
Enpp2
Gabrd
Gad1
Gap43
Id2
Lamp5
Neurod6
Nov
Nr4a2
Nrn1
Pde1a
Plp1
Reln
Rgs12
Satb1
Scg2
Slc6a1
Sulf2
Tac1
Zcchc12
A C E H I L O S T

Figure S9

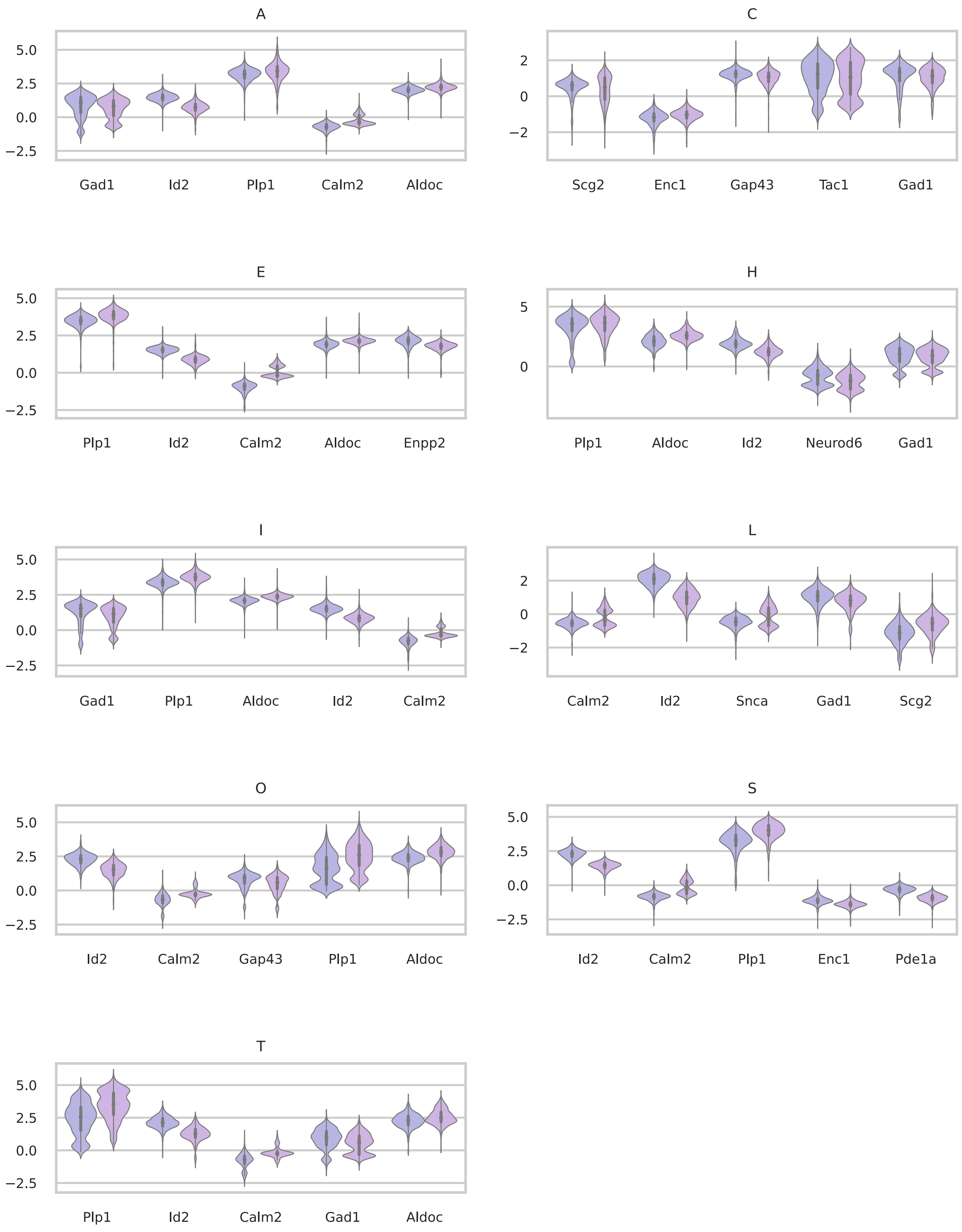
A
Gad1
Id2
Plp1
Calm2
Aldoc
C
Scg2
Enc1
Gap43
Tac1
Gad1
E
Plp1
Id2
Calm2
Aldoc
Enpp2
H
Plp1
Aldoc
Id2
Neurod6
Gad1
I
Gad1
Plp1
Aldoc
Id2
Calm2
L
Calm2
Id2
Snca
Gad1
Scg2
O
Id2
Calm2
Gap43
Plp1
Aldoc
S
Id2
Calm2
Plp1
Enc1
Pde1a
T
Plp1
Id2
Calm2
Gad1
Aldoc

Figure S10

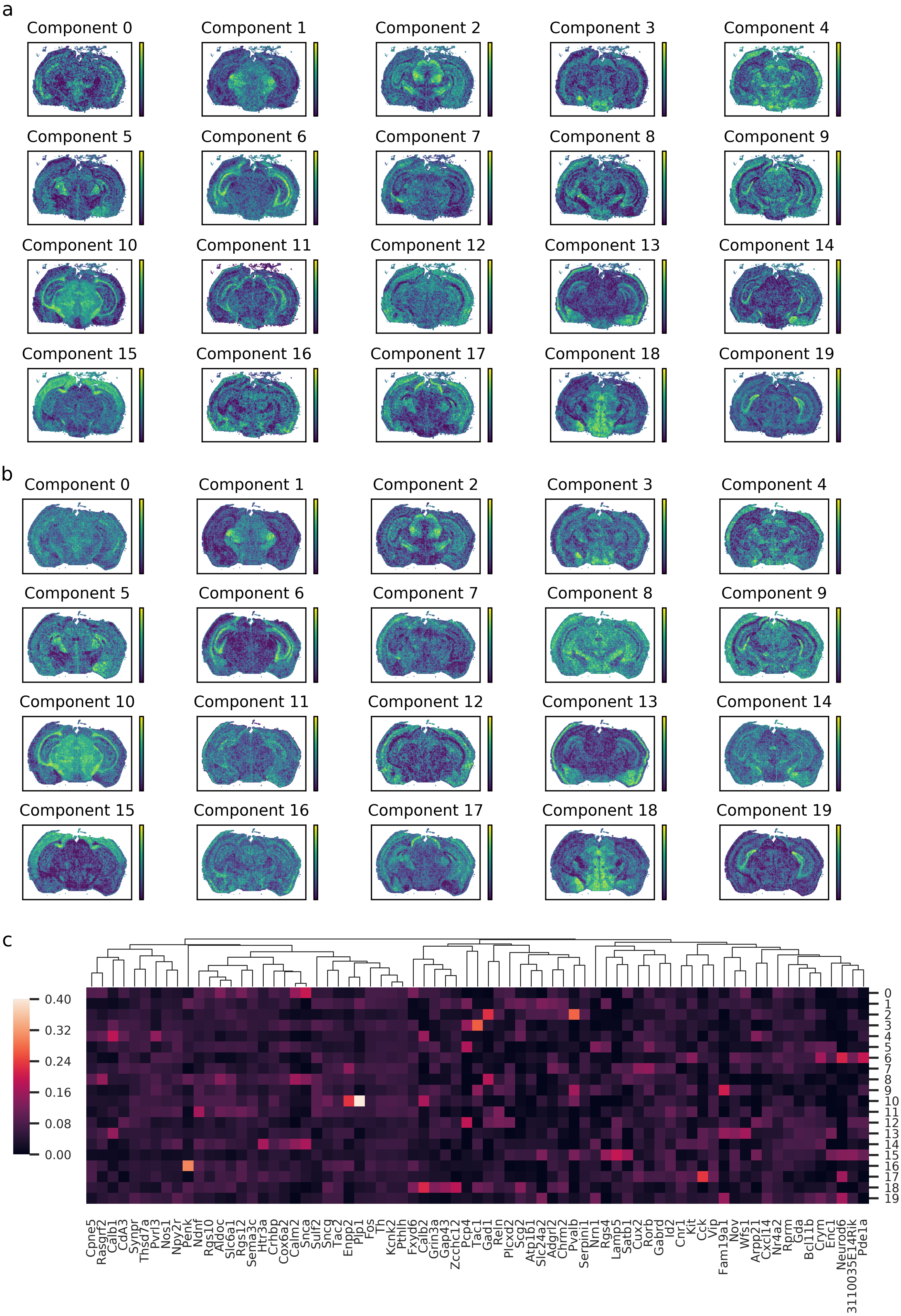
a
Component 0
Component 1
Component 2
Component 3
Component 4
Component 5
Component 6
Component 7
Component 8
Component 9
Component 10
Component 11
Component 12
Component 13
Component 14
Component 15
Component 16
Component 17
Component 18
Component 19
b
Component 0
Component 1
Component 2
Component 3
Component 4
Component 5
Component 6
Component 7
Component 8
Component 9
Component 10
Component 11
Component 12
Component 13
Component 14
Component 15
Component 16
Component 17
Component 18
Component 19
c
0.40
0.32
0.24
0.16
0.08
0.00
0
1
2
3
4
5
6
7
8
9
10
11
12
13
14
15
16
17
18
19
Cpne5
Rasgrf2
Calb1
CdA3
Synpr
Thsd7a
Pvrl3
Nos1
Npy2r
Penk
Ndnf
Rgs10
Aldoc
Slc6a1
Rgs12
Sema3c
Htr3a
Crhbp
Cox6a2
Calm2
Snca
Sulf2
Sncg
Tac2
Enpp2
Plp1
Fos
Th
Kcnk2
Pthlh
Fxyd6
Calb2
Grin3a
Gap43
Zcchc12
Pcp4
Tac1
Gad1
Reln
Plcxd2
Scg2
Atp1b1
Slc24a2
Adgrl2
Chrm2
Pvalb
Serpini1
Nrn1
Rgs4
Lamp5
Satb1
Cux2
Rorb
Gabrd
Id2
Cnr1
Kit
Cck
Vip
Fam19a1
Nov
Wfs1
Arpp21
Cxcl14
Nr4a2
Rprm
Gda
Bcl11b
Crym
Enc1
Neurod6
3110035E14Rik
Pde1a

Figure S11

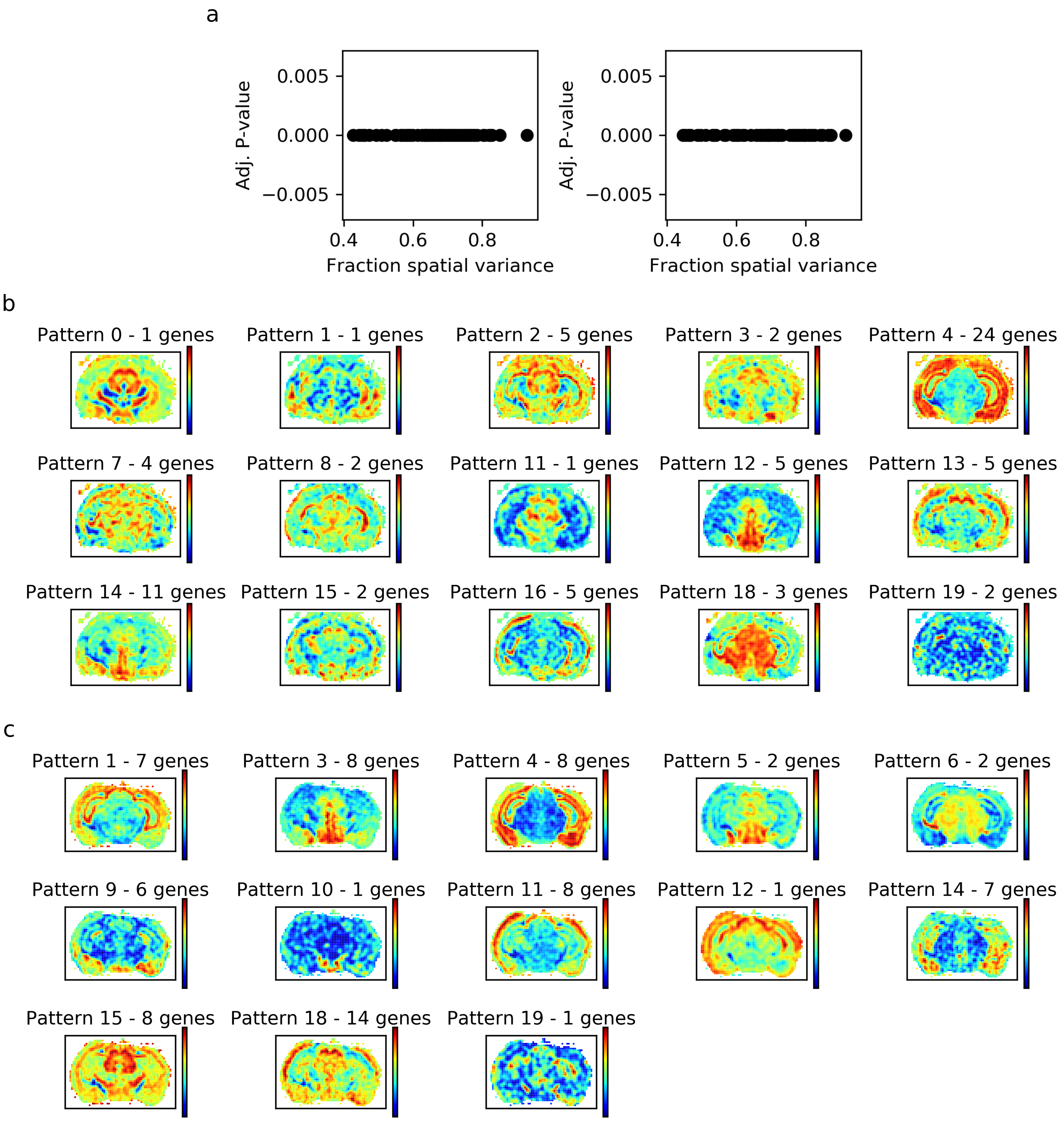
a
Adj. P-value
0.005
0.000
−0.005
0.4
0.6
0.8
Fraction spatial variance
Adj. P-value
0.005
0.000
−0.005
0.4
0.6
0.8
Fraction spatial variance
b
Pattern 0 - 1 genes
Pattern 1 - 1 genes
Pattern 2 - 5 genes
Pattern 3 - 2 genes
Pattern 4 - 24 genes
Pattern 7 - 4 genes
Pattern 8 - 2 genes
Pattern 11 - 1 genes
Pattern 12 - 5 genes
Pattern 13 - 5 genes
Pattern 14 - 11 genes
Pattern 15 - 2 genes
Pattern 16 - 5 genes
Pattern 18 - 3 genes
Pattern 19 - 2 genes
c
Pattern 1 - 7 genes
Pattern 3 - 8 genes
Pattern 4 - 8 genes
Pattern 5 - 2 genes
Pattern 6 - 2 genes
Pattern 9 - 6 genes
Pattern 10 - 1 genes
Pattern 11 - 8 genes
Pattern 12 - 1 genes
Pattern 14 - 7 genes
Pattern 15 - 8 genes
Pattern 18 - 14 genes
Pattern 19 - 1 genes

Figure S12

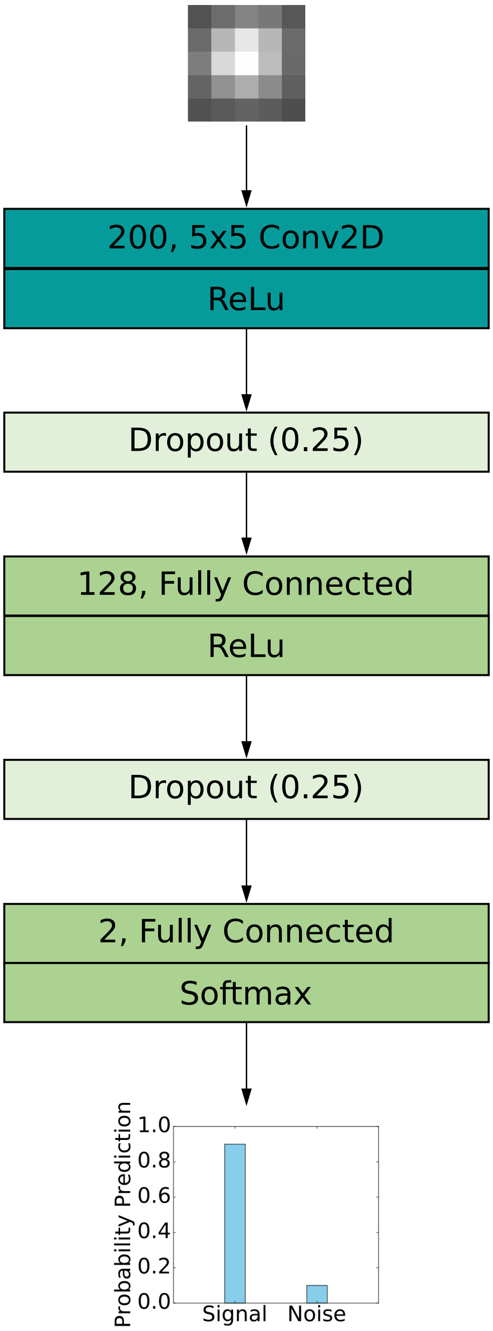
200, 5x5 Conv2D
ReLu
Dropout (0.25)
128, Fully Connected
ReLu
Dropout (0.25)
2, Fully Connected
Softmax
Probability Prediction
1.0
0.8
0.6
0.4
0.2
0.0
Signal
Noise

Table S1a

Pattern 0	Gad1
Pattern 1	Rgs12
Pattern 2	Adgrl2, Atp1b1, Kit, Serpini1, Pvalb
Pattern 3	Reln, CdA3
Pattern 4	Calm2, Rgs4, Pde1a, Nrn1, Nov, Neurod6, Lamp5, Gda, Gabrd, Crym, Crhbp, Cck, Bcl11b, Arpp21, 3110035E14Rik, Vip, Rprm, Nr4a2, Htr3a, Enc1, Cox6a2, Cnr1, Satb1, Snca
Pattern 7	Plcxd2, Sema3c, Sulf2, Slc24a2
Pattern 8	Aldoc, Ndnf
Pattern 11	Chrm2
Pattern 12	Calb2, Scg2, Sncg, Tac1, Zcchc12
Pattern 13	Calb1, Cxcl14, Rorb, Thsd7a, Id2
Pattern 14	Fos, Grin3a, Nos1, Penk, Rasgrf2, Rgs10, Cpne5, Fxyd6, Gap43, Th, Wfs1
Pattern 15	Pcp4, Pthlh
Pattern 16	Fam19a1, Pvrl3, Tac2, Cux2, Npy2r
Pattern 18	Enpp2, Plp1, Slc6a1
Pattern 19	Kcnk2, Synpr

Table S1b

Pattern 1	Fos, Kit, Nrn1, Cck, Neurod6, Satb1, Slc24a2
Pattern 3	Nos1, Penk, Th, Calb2, Fxyd6, Gap43, Sncg, Zcchc12
Pattern 4	Rprm, 3110035E14Rik, Bcl11b, Crym, Enc1, Gda, Nov, Pde1a
Pattern 5	Scg2, Tac1
Pattern 6	Enpp2, Plp1
Pattern 9	CdA3, Npy2r, Synpr, Calb1, Cox6a2, Wfs1
Pattern 10	Tac2
Pattern 11	Calm2, Fam19a1, Arpp21, Cux2, Gabrd, Lamp5, Rgs4, Vip
Pattern 12	Id2
Pattern 14	Cnr1, Crhbp, Htr3a, Nr4a2, Snca, Cxcl14, Rgs12
Pattern 15	Adgrl2, Atp1b1, Serpini1, Sulf2, Chrm2, Gad1, Pcp4, Pvalb
Pattern 18	Aldoc, Cpne5, Grin3a, Kcnk2, Pthlh, Rasgrf2, Reln, Rgs10, Sema3c, Slc6a1, Thsd7a, Plcxd2, Pvrl3, Rorb
Pattern 19	Ndnf
